# Neurosyphilis with positive anti-N-methyl-D-aspartate receptor antibody: a case report

**DOI:** 10.3389/fneur.2023.1164605

**Published:** 2023-05-18

**Authors:** Zhu Sha, Shi Jing, Gao Feng, Hao Hongjun, Liu Xianzeng

**Affiliations:** ^1^Department of Neurology, Peking University International Hospital, Beijing, China; ^2^Department of Radiology, Peking University First Hospital, Beijing, China

**Keywords:** neurosyphilis, encephalitis, anti-N-methyl-D-aspartate receptor (anti-NMDAR), epilepsy, case report

## Abstract

A case of neurosyphilis with a positive anti-N-methyl-D-aspartate receptor (NMDAR) antibody was reported. A 54-year-old man who presented with acute memory deficits was admitted to our hospital. Acute ischemic stroke (AIS) was initially considered, and he was prescribed intravenous thrombolysis with recombinant tissue-type plasminogen activator (rt-PA). However, the intermittent onset of episodic memory and orientation disorder still occurred. No diffusion restriction was indicated by magnetic resonance imaging (MRI), and subclinical seizures were frequently found by electroencephalogram (EEG). Rapid plasma reagin (RPR) test of serum showed positive results for syphilis. Analysis of cerebrospinal fluid (CSF) revealed elevated leukocyte count and protein level. RPR test, *Treponema pallidum* particle agglutination (TPPA) assay, and *Treponema pallidum* antibody (TP-Ab) in CSF showed positive results, and the anti-NMDAR antibodies were positive in CSF and serum. Finally, the patient was diagnosed with neurosyphilis with a positive anti-NMDAR antibody. The clinical symptoms were improved, and the leukocyte count in CSF was reduced after treatment with intravenous penicillin G and levetiracetam. This case suggests that in cases with positive results for neurosyphilis and NMDAR antibodies, the proper treatment has to be decided based on all of the available clinical and diagnostic testing data.

## Introduction

*Treponema pallidum* is one of the most common causes of sexually transmitted infections. Infection with *Treponema pallidum* is most commonly referred to as syphilis with modifiers to denote the phase of the disease or infection manifestations. Neurosyphilis is an infection of the central nervous system (CNS) caused by *Treponema pallidum*, which may occur at any stage of the infection (1). The incidence of syphilis declined after the introduction of penicillin. The incidence rate has shown an upward trend since the 2000's, and especially with the increase in the prevalence of acquired immunodeficiency syndrome (AIDS) and immunodeficiency, the number of patients with neurosyphilis has gradually risen (2, 3). In China, the incidence of syphilis ranks third after viral hepatitis and tuberculosis among infectious diseases. The upward trend was aligned with that of neurosyphilis (4).

Autoimmune encephalitis (AE) generally refers to a type of encephalitis mediated by autoimmunity. At present, the prevalence of AE accounts for about 10~20% of encephalitis, and its clinical manifestations include acute or subacute cognitive impairment, epileptic seizures, mental disorders, and a variety of motor disorders (5). Anti-NMDAR encephalitis is a relatively common form of AE and one of the most completely described forms of AE; however, we do not have accurate estimates for the incidence of NMDAR encephalitis or all other forms of AE. Initially, anti-NMDAR encephalitis was thought to be associated with malignancies, and it was later found to be common after viral infections (6).

Neurosyphilis may be latent and asymptomatic or accompanied by a variety of signs and non-specific clinical symptoms, mimicking several types of neurological and psychiatric diseases. To date, neurosyphilis with a positive anti-NMDAR antibody has been rarely reported. The present study aimed to report such a case.

## Case presentation

A 54-year-old man was referred to the emergency department by his son, who complained of his father's memory impairment for 1 h, presenting that he could not find the way home and answer questions correctly, without movement and sensory disorders. Before the attack, he took a walk near his home as usual. His medical history included hypertension and diabetes. He suffered from a stroke 8 years ago, leaving over mild weakness of the right lower limb and mild memory decline, and was competent for agricultural work. Five years ago, he suffered a stroke again, presenting with increased weakness in his right lower limb, which gradually returned to baseline levels. However, his memory and executive function gradually decreased, and he was still able to pursue simple farm work, take care of himself, accompanied by personality change, and became irritable. He was taking aspirin regularly after the stroke. The patient had a history of smoking. Physical examination revealed disorientation, slow reaction, and lack of cooperation. The patient had an acute onset, and the cranial computed tomography (CT) showed multiple areas of signal abnormality consistent with encephalomalacia as a consequence of remote cerebral infarction. Acute ischemic stroke (AIS) was considered and he was prescribed intravenous thrombolysis with recombinant tissue-type plasminogen activator (rt-PA). After thrombolytic therapy, the patient regained his directional ability about half an hour later. and neurological examination indicated slow response, calculation decline, right-left agnosia, memory decline, 5-/5 strength of the right lower limb, and right Babinski's sign-positive. The other parameters of the neurological examination were normal. Mini-Mental State Exam (MMSE) score was 15/30 (middle school education level).

**Figure 1:**
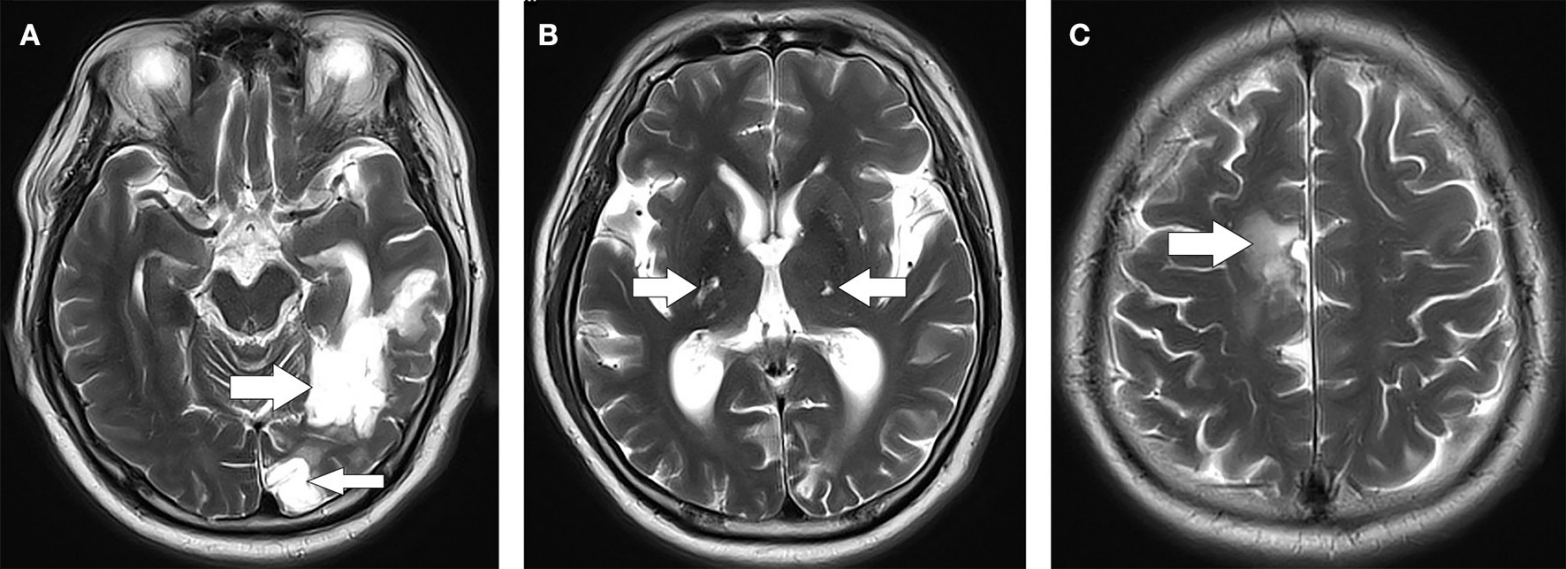
Axial section of T2 brain MRI. **(A)** Cerebral softening lesions in the left temporal-occipital lobe (indicated by arrows). **(B)** Multiple lacunar infarctions in the bilateral basal ganglia (indicated by arrows). **(C)** Cerebral softening lesions in the right corona radiata (indicated by arrow). MRI, magnetic resonance imaging.

The patient was subsequently admitted to the neurology ward for further treatment. In addition, CT angiography (CTA) of the head and neck showed no obvious stenosis. Non-enhanced magnetic resonance imaging (MRI) of the brain revealed signal abnormality consistent with his history of ischemic stroke, including encephalomalacia and chronic lacunes (Figure 1). However, there was no evidence of restricted diffusion as it would be expected that his acute symptoms were caused by an AIS. After admission to the ward, the patient had recurrent episodic disorientation. It lasted for more than 10–30 min for each attack, and he could not recall it afterward. Therefore, non-convulsive status epilepticus (NCSE) was suspected. During Electroencephalogram (EEG) monitoring, the patient did not experience an acute disorientation attack. An EEG showed that more than 40 subclinical seizures were recorded within 16 h.

The routine blood parameters, liver function, kidney function, electrolyte, coagulation profile, anti-nuclear antibodies, anti-neutrophil cytoplasmic antibodies, rheumatoid factors, thyroid function, anti-thyroglobulin antibodies, anti-thyroid peroxidase antibodies, serum tumor markers, and serological testing for hepatitis B and human immunodeficiency virus (HIV) were normal. Fasting blood glucose was 16.5 mmol/L. Triglyceride level was 3.31 mmol/L, and low-density lipoprotein cholesterol was 3.1 mmol/L. Rapid plasma reagin (RPR) testing indicated positive results for syphilis (serum RPR titer of 1:32). The patient's son supplemented his medical history. Syphilis had been identified when his father suffered from a stroke 8 years ago. He received standardized treatment for syphilis. He showed progressive cognitive impairment, personality change, and seizures on admission; therefore, neurosyphilis was considered. A lumbar puncture was performed, and normal opening pressure was indicated. The count of leukocytes in cerebrospinal fluid (CSF) increased to 117/μl, of which 97% were monocytes, and red blood cells (RBCs) were 2/μl. The glucose level in CSF was 5.9 mmol/L, and the protein level in CSF was elevated (829.19 mg/L). RPR test, *Treponema pallidum* particle agglutination (TPPA) assay, and *Treponema pallidum* antibody (TP-Ab) in CSF showed positive results. The oligoclonal immunoglobulin G (IgG) bands (OCB, type II) were positive in CSF, while they were negative in serum. In addition, the patient was tested for AE antibodies by cell-based assay (CBA), in which the anti-NMDAR antibodies were positive in CSF and serum, and the titers in CSF and serum were 1:20 and 1:10, respectively. Hu, Yo, and Ri antibodies were not detected in both serum and CSF.

Therefore, the patient was finally diagnosed with neurosyphilis with anti–NMDAR antibodies. Intravenous penicillin G (24 million units/d for 3 weeks) and levetiracetam (up to 500 mg twice daily) were given. Simultaneously, antiplatelet drugs and statins were prescribed for secondary prevention and treatment of cerebrovascular diseases. In addition, the blood glucose and blood pressure of the patient were well controlled. After the above-mentioned treatment, the patient's paroxysmal disorientation and cognitive impairment were alleviated. After 3 weeks of treatment with penicillin, we reviewed the patient's EEG, MMSE, and CSF tests. Reexamination of the EEG showed no clinical or subclinical seizures within 16 h. MMSE increased to 19/30 points. The protein level in CSF is 831.37 mg/L. The count of leukocytes in CSF was reduced to 15/μl, with 5 RBCs /μl. In addition, OCB in CSF remained positive and the anti-NMDAR antibody titer remained unchanged. After 5 weeks, the patient was positive for RPR with a serum titer of 1:8.

## Discussion

A case of neurosyphilis with an anti-NMDAR antibody was reported in the present study. The patient presented with acute and non-specific changes in mental status manifested as memory loss and disorientation. AIS was initially considered, and rt-PA was prescribed, while recurrent disorientation was continued after admission to the ward. The brain MRI did not show restricted diffusion, and multiple non-convulsive and subclinical seizures were recorded by EEG. Thus, the diagnosis of NCSE was considered to be more likely than a diagnosis of AIS evolving to NCSE. The patient had a history of syphilis, with clinical manifestations of epilepsy, dementia, and RPR, TPPA, and TP-Ab in CSF were positive. Therefore, he was diagnosed with neurosyphilis. After 3 weeks of intravenous penicillin G and antiepileptic therapy, the symptoms were relieved, and the leukocyte count in CSF was lowered down.

Neurosyphilis is a slow-progressing, destructive infection of the brain and spinal cord. The incidence of neurosyphilis was estimated to be 0.47–2.1 per 100,000 people (7, 8). The clinical stages of syphilis include early syphilis, late syphilis, and neurosyphilis. Early neurosyphilis occurs several months to several years after infection, and it is typically manifested as meningitis or meningeal vascular disease, while late neurosyphilis occurs several years to several decades after infection, which is characterized by general paresis, including progressive dementia, psychiatric syndromes, personality change, manic delusions, tremor, and dysarthria (9). A study on 286 neurosyphilis patients found that general paralysis of the insane was the most common type of neurosyphilis (49%), followed by syphilitic meningitis (22%), meningovascular, and tabetic types (12%) (10). Multiple brain softening and infarct focus may be related to meningovascular neurosyphilis, however, they may also be related to atherosclerosis because of hypertension and diabetes. Therefore, the etiological diagnosis of cerebral infarction is still a clinical challenge. The patient had cognitive impairment after cerebral infarction. Moreover, the brain MRI showed temporal lobe infarction and vascular dementia was also indicated. However, the patient's cognitive impairment gradually worsened, and neurosyphilitic dementia was also considered. Thus, the patient was diagnosed with late neurosyphilis. A study on 120 neurosyphilis patients found that 25% of patients had seizures, among which half had generalized seizures, most had focal seizures, and few patients had status epilepticus (11). To the best of our knowledge, neurosyphilis has rarely been reported as NCSE. The changes of the patient in an acute mental state, manifested as memory loss and disorientation, can also be explained by NCSE, which further confirms the diversity of clinical manifestations of neurosyphilis.

Anti-NMDAR encephalitis was first described in 2005 as a clinical syndrome of acute episode psychosis, and a progressive while treatable encephalopathy. The disease mainly covers five distinct stages: prodromal phase, psychotic phase, unresponsive phase, hyperkinetic phase, and recovery phase (12–14). It may present with psychosis, memory deficits, seizures, dyskinesia, involuntary movements, decreased level of consciousness, and autonomic instability. Seizures are a common manifestation of this disease, and they were found in 76–82% of patients (15, 16). Extreme delta brush is a specific EEG pattern identified in 30% of patients with anti-NMDAR encephalitis (17). The mechanisms that may trigger AE include tumors, infections, or cryptogenic factors (18). Virus-mediated cerebral tissue damage may lead to antigen exposure that triggers the development of anti-neuronal antibodies (19). Therefore, it is reasonable to speculate that *T. pallidum* infects the CNS, resulting in the exposure of antigens that may produce anti-NMDAR antibodies.

To date, it has been reported that neurosyphilis is characterized by marginal lobe encephalitis, and studies have found the coexistence of AE antibodies with neurosyphilis (20–23). Furthermore, cases of neurosyphilis with anti-NMDAR antibodies were searched in PubMed with the target words “neurosyphilis” or “syphilis” and “anti-N-methyl-D-aspartate Receptor antibody” or “NMDA” or “anti-NMDAR encephalitis.” A total of four cases (three reports) were matched (23–25), and all the cases were men aged over 30. Table 1 summarizes the characteristics of these four cases. Case 1 showed progressively reduced vision in both eyes, and the symptom was alleviated by penicillin. Case 2 presented progressive attention and memory impairment and symptoms were relieved after the use of penicillin combined with steroid hormone and intravenous immunoglobulin (IVIG). Case 3 presented with progressive psychobehavioral abnormalities and tonic-clonic seizures, which are ineffective after receiving IVIG. Symptoms are relieved after switching to penicillin. The condition of case 4 was worsened after the application of ceftriaxone, and symptoms were relieved after switching to steroid hormone and IVIG. The patient in our study started with NCSE and the anti-NMDAR antibody in CSF was positive, but AE was not considered. Because clinical presentation, course, imaging findings, CSF, and laboratory testing results of this patient could be explained by syphilis and seizure, and his symptoms were improved after treatment of penicillin and levetiracetam without immunomodulatory therapies, although neurosyphilis might induce immune abnormalities and lead to the generation of anti-NMDAR antibodies, we believe that it might not cause disease to this patient, and might be existed as a bystander. Case 1 and Case 3 also confirmed this viewpoint. Case 2 was treated with both penicillin and immunomodulatory therapy, and it is unclear which medication improved the symptoms. The symptoms of Case 4 were worsened after the application of ceftriaxone, but symptoms were improved after switching to hormones and IVIG, which seems contradictory. The roles of anti-NMDAR antibodies in neurosyphilis may be bystanders or pathogenic factors, which requires further theoretical and clinical research to clarify. In a word, in cases with positive results for syphilis/neurosyphilis and NMDAR, the proper treatment has to be decided based on the available clinical and diagnostic testing data. When IgG specific for the GluN1 subunit of the NMDAR is found in both serum and CSF, this antibody should not be considered as the cause of a person's neurologic illness unless a compatible clinical syndrome is available and alternative causes of encephalitis have been ruled out. In cases for which a treatable infection is also found, the risks and benefits of concomitant anti-microbial therapy and immunotherapy need to be evaluated.

**Table 1:** Cases of neurosyphilis with positive anti-NMDAR antibody.

	**Age**	**Gender**	**Symptoms**	**Brain MRI**	**EEG**	**CSF**	**Treatment and outcome**
Case 1 (24)	37	Male	Progressively reduced vision of both eyes for 6 months	A focal slightly high FLAIR signal on the right frontal lobe and low T2 signal adjacent to the right cornu posterious ventriculi lateralis	Nil	Leukocyte:6/mm3 Protein:1.237 g/L OB: positive	Penicillin G: improved
Case 2 (24)	39	Male	Progressive attention and memory impairments	Enlargement of lateral ventricles and the third ventricle, and focal high T2/FLAIR signal abnormality involving bilateral temporal lobe, and corona radiate	Nil	Leukocyte: normal Protein: normal OB: positive	Penicillin G + pulsed methylprednisolone + IVIG: improved
Case 3 (23)	35	Male	Abnormal mental and behavior changes for 3 months. Tonic-clonic seizures on admission	Normal	Intermittent frontal slowness	Leukocyte:52/Ul, 96% were monocytes Protein: 143 mg/dL OB: No description	IVIG: inefficiency Penicillin G: improved
Case 4 (25)	32	Male	Cognitive decline, diplopia and walking instability for 6 months	Symmetrical abnormal signals in the pons, midbrain, and bilateral basal ganglia	Normal	Leukocyte: normal Protein: normal OB: positive	Ceftriaxone: worsen Pulsed methylprednisolone + IVIG: improved

MRI, magnetic resonance imaging; FLAIR, Flow attenuated inversion recovery; EEG, electroencephalogram; CSF, cerebrospinal fluid; IVIG, intravenous immunoglobulin.

## Data availability statement

The raw data supporting the conclusions of this article will be made available by the authors, without undue reservation.

## Ethics statement

Written informed consent was obtained from the individual(s) for the publication of any potentially identifiable images or data included in this article.

## Author contributions

ZS collected data and drafted the manuscript. SJ consulted the relevant literature. GF and HH contributed to the guidance of the report. LX contributed to the guidance of the research and review of the manuscript. All authors contributed to the article and approved the submitted version.
